# When Two Worlds Collide: The Contribution and Association Between Genetics (APOEε4) and Neuroinflammation (IL-1β) in Alzheimer’s Neuropathogenesis

**DOI:** 10.3390/cells14151216

**Published:** 2025-08-07

**Authors:** Jagadeesh Narasimhappagari, Ling Liu, Meenakshisundaram Balasubramaniam, Srinivas Ayyadevara, W. Sue T. Griffin

**Affiliations:** 1 Donald W. Reynolds Department of Geriatrics, Reynolds Institute on Aging, University of Arkansas for Medical Sciences, Little Rock, AR 72205, USA; njagadeesh@uams.edu (J.N.); LiuLing@uams.edu (L.L.); mbalasubramaniam@uams.edu (M.B.); ayyadevarasrinivas@uams.edu (S.A.); 2 Central Arkansas Veterans Healthcare System, Little Rock, AR 72205, USA

**Keywords:** APOEε4, Interleukin-1β, Neuroinflammation, autophagy

## Abstract

**Introduction:** Here we consider the collision of a genetic factor and an essential instigator in Alzheimer’s neuropathogenesis: (i) the Alzheimer’s gene (APOEε4), which downregulates lysosomal autophagy and induces synthesis of (ii) the instigator, interleukin-1β (IL-1β), which drives synthesis of βAPP for Aβ plaques and of MAPKp38 for phosphorylation of tau for formation of neurofibrillary tangles (NFTs), the two cardinal features of AD. **Methods:** RT-PCR, immunoblotting and immunohistochemistry techniques were used to assess the levels of IL-1β and its signaling cascade in ADε4,4, ε3,3, and age-matched controls (AMC3,3) in hippocampal regions of the brain. **Results:** IL-1β and its downstream signaling proteins TLR-2, MyD88, NFκB, COX-1, and COX-2 were greater in tissues from ADε4,4 than ADε3,3 or AMC3,3. Cathepsin B, D, and L levels, which play a pivotal role and are necessary for lysosomal autophagy, were lower in ADε4,4 than in ADε3,3 or AMC ε3,3. IL-1β and its downstream signaling cascade TLR-2, MyD88, NFκB, COX-1, and COX-2 expression levels were high in SH-SY5Y and T98G cells transfected with APOεE4. **Conclusions:** APOEε4 causes Alzheimer’s by downregulating autophagy, thus inducing IL-1β for Aβ plaque and neurofibrillary tangle formation.

## 1. Introduction

As always, untoward genetics play “a”, or perhaps “the,” prominent role in disease. This is true in Alzheimer’s disease (AD), as illustrated in (i) specific rare familial genetic anomalies that account for approximately two percent of all Alzheimer’s cases; i.e., such genetic variants result in the clinical manifestations of dementia as well as the two neuropathognomic features of AD, viz., Aβ plaques that lie adjacent to neurons, many of which contain neurofibrillary tangles [1,2,3]. Specific numbers of such Aβ plaques and tangle-bearing neurons in specified brain regions are required for a diagnosis of dementia of the Alzheimer’s type. (ii) Down’s syndrome, with its triplication of chromosome 21 and its virtually certain early manifestation of the neuropathological features of AD in middle age [4], including Aβ plaques and neurofibrillary tangles, provides another example of the collision between untoward genetics and neuroinflammation. To a much greater extent than either rare genetic anomalies or Down’s syndrome is (iii) inheritance of APOEε4, the “Alzheimer Gene,” which Mahley estimates is present in 50–65 percent of those with AD—now estimated to number 7 million people in the US—clearly implicating inheritance of APOEε4 as a major “causative factor” in Alzheimer’s neuropathogenesis [5]. Alzheimer’s dementia, neuropathologically diagnosed by a given number of amyloid beta (Aβ) plaques outside neurons and neurofibrillary tangles (NFTs) within them in specific brain regions, is the most common form of dementia worldwide and is predicted to reach 139 million globally and to almost double from the current estimate of 7 million to a predicted number of 13 million in the US by 2050 [6]. In our previous reports, this seemed to be the case as a result of ApoE4, the protein product of the APOEε4 gene, entering the nucleus, binding to CLEAR DNA (the Coordinated Lysosomal Expression and Regulation site on DNA), and in this way blocking the production of three proteins, viz., sequestosome p62, LC3B, and LAMP2, which are necessary for lysosomal autophagy [7]. The resulting downregulation of lysosomal autophagy, with its thwarted cellular waste clearance, favors aggregate formation as evidenced by the presence of more Aβ plaques and neurofibrillary tangles in APOEε4 carriers [8,9]. However, it is especially important to point out that the presence and proliferation of both of these cardinal features of Alzheimer’s is wholly dependent on the pluripotent neuroinflammatory cytokine IL-1β, which we dub here as the essential instigator, as the driver of the genesis of both Aβ plaques and neurofibrillary tangles [10], as it induces the synthesis of βAPP, the precursor of Aβ, which is the seed protein for Aβ plaques in APOEε4 carriers and is further elevated at both mRNA and protein levels [10]. Evidence for this is also provided in Down’s syndrome, with its frank appearance of Alzheimer’s Aβ plaques and neurofibrillary tangle-bearing neurons in early middle age [11], a pathological manifestation that we showed was presaged by dramatic neonatal overexpression of IL-1β that continued into adulthood [12]. It has become increasingly clear that IL-1β is by far the principal actor in the development of AD in response to any and all neuronal insults, including direct acute brain injury as in contact sports and motor vehicle accidents, or sustained insults, including the excess neuronal activity in epilepsy [13], especially in the context of the overlay of inheritance of the Alzheimer’s gene, which we showed manifests as frank mature Alzheimer’s plaques even in childhood, as noted in a 10-year-old with numerous, frank Aβ plaques [13]. As an example and explanation of the neuroinflammatory process, Barger and Griffin and their colleagues showed that IL-1β [14] in response to glutamate-induced neuronal stress results in increased neuronal expression of β-amyloid precursor protein (βAPP) and release of secreted APPα (sAPPα) [14], thus furthering microglial activation and release of IL-1β and, in this way, increasing the levels of Aβ and Aβ plaque burden, creating a self-sustaining cycle of neuronal stress and more IL-1β and Aβ plaques [15].

In addition to Aβ plaque production, IL-1β is also responsible for the other characteristic feature of AD, viz., neurofibrillary tangles. This is the result of IL-1β induction of both the synthesis of MAPK-p38 and the elevation of its activation state for phosphorylation of tau, which is a necessary step in neurofibrillary tangle formation [15]. It is important to note that the presence of IL-1β-driven increased numbers of both Aβ plaques adjacent to neurons and neurofibrillary tangles within them create further neuronal stress, making it logical to conclude that, if unchallenged, a self-repeating cycle of IL-1β-driven accumulation of Aβ plaques and neurofibrillary tangles is inevitable [14]. Taken together, these facts implicate IL-1β as necessary for the development of the two cardinal diagnostic features of AD. Further, the inheritance of APOEε4 alleles in 50–65 percent of all these cases [2] has the added burden of a diminution in lysosomal autophagy, which, together with a myriad of other untoward features of such APOE4 inheritance, allows for the hypothesis that, in collision, these two driving factors—inheritance of the Alzheimer’s gene, together with IL-1β-engendered neuroinflammation—may be said to account for all or virtually all cases of AD. Considering this, the present study aimed to study the role of IL-1β and its signaling cascade in hippocampus tissues from eighteen brains obtained at autopsy from six neurologically normal individuals (AMCs, aged 61–81 years) and twelve Alzheimer’s patients (six were APOE ε3,3, and six were APOE ε4,4).

## 2. Methods

### 2.1. Patients and Specimens

Tissue samples used in this study were acquired at the time of autopsy at the University of Arkansas for Medical Sciences (UAMS). All the samples collected were evaluated neuropathologically by one of us (REM). Patients classified as age-matched controls (AMCs) were clinically represented as non-demented and were evaluated according to the CERAD (Consortium to Establish a Registry for Alzheimer’s Disease) criteria as lacking AD pathology and by Braak staging as 1 or 0 [16,17]. All AD cases were allotted CERAD scores of C (definite Alzheimer’s) and Braak stages IV-VI. All the collected samples were stored in our UAMS Brain Bank either as snap-frozen 3-D dissected regions or as formalin-fixed, paraffin-embedded blocks. Eighteen brains were collected at autopsy from six individuals without neurological disease (AMCs, aged 61–81 years, with the APOE ε3,3 genotype) and twelve Alzheimer’s patients (AD, aged 45–95 years; six patients had the APOE ε4,4 genotype, and the other six patients had the APOE ε3,3 genotype). All tissues used in the study were neuropathologically assessed, and the diagnoses of AD or AMCs were made according to the National Institute of Aging—Reagan guidelines.

Flash-frozen tissues samples from hippocampi stored at −80 °C were pulverized using a mortar and pestle, cooled on dry ice, and used for RNA and protein extraction.

### 2.2. Ethics, Consent, and Permissions

Human tissues used in this study were collected at autopsy for reasons other than for this study and were judged as exempt from federal regulations on human subjects via exemption 4, with Human Subject Assurance Number 00001119, by the University, in strict accordance with the recommendations of the US National Institutes of Health Guide for the Care and Use of Laboratory Animals. All protocols used in the study were approved (approval number, a3063-01) by the Institutional Animal Care and Use Committee (IACUC), and all efforts were made to minimize pain and suffering.

### 2.3. Tissue Culture and Maintenance

Human neuroblastoma (SH-SY5Y) and glioblastoma cells (T98G) cell lines were transfected with APOEε3 and APOEε4 genes, generated as described previously by Wang et al. [18]. SH-SY5Y and T98G cells, expressing either APOEε3 or APOEε4 genes, were seeded in T75 culture flasks, grown on Dulbecco’s Modified Eagle’s medium (DMEM) (Cat. No: 119950065, Thermo Fisher Scientific, Waltham, MA, USA), which was supplemented with 10% fetal bovine serum (FBS), for 48 h to reach confluency (Cat. No: 16000044, Thermo Fisher Scientific).

### 2.4. Human Hippocampal Immunohistochemistry

Hippocampal tissue blocks used in the study (3 from each group) were sectioned at a thickness of 7 μm, deparaffinized in xylene, rehydrated in serial dilutions of ethanol to water, and washed with PBS + 0.1% Tween 20. After deparaffinization, endogenous peroxidase activity was blocked with 3% H_2_O_2_. Antigen retrieval was performed in citrate buffer (100 °C for 30 min), and the endogenous background activities were blocked in normal rabbit serum, according to instructions in the Vector Elite Goat Kit (PK-6105), CA, USA for 20 min. Sections were incubated in IL-1β antibody at 1:200 overnight at 4 °C. After primary antibody incubation, the sections were washed three times with PBST, and the sections were incubated in rabbit ImmPRESS peroxidase reagent for 30 min, followed by a 2 min incubation with Deep Space Black Chromagen solution (BioCare Medical, Concord, CA, USA) to quench lipofuscin interference. After serial dehydration, the sections were cover-slipped with Micromount.

### 2.5. Image Analysis

Images were taken on a Nikon Eclipse E600 Microscope (Nikon, Tokyo, Japan) with a Cool Snap ES camera. Quantitative immunofluorescence was performed with the same exposure times among all samples for the same measurement. Data were analyzed using an unpaired *t*-test, and values were considered significantly different when the two-tailed *p* value was ≤0.05. Results are expressed as mean ± SEM.

### 2.6. RT-PCR Amplification

IL-1β induces a TLR-MYD88-NF-κB downstream signaling cascade in Parkinson’s disease, leading to the upregulation of α–synuclein [19]. In the present study, to evaluate the IL-1β-induced TLR-MYD88-NF-κB downstream signaling cascade, the following genes (Table 1) were used. Flash-frozen hippocampal tissues from 18 brains were homogenized in a mortar and pestle and cooled on dry ice. Total RNA was extracted from human brain samples of AMC, AD 3,3, and AD 4,4 patients (six each) using a Qiagen RNA RNeasy Plus Mini extraction kit (#74134), following the manufacturer’s instructions. The quality and quantity of the extracted RNA was estimated using an Agilent bioanalyzer. All the RT reactions were performed on equal amounts of RNA from the tissue samples using single-step RT-PCR reagents (#11736059 ThermoFisher, Waltham, MA, USA). The sequences of primers used in the study were made and purchased from IDT as depicted in the table below.

### 2.7. Western Blot

Flash-frozen hippocampus tissues from 18 brains were homogenized using a mortar and pestle and cooled on dry ice. Proteins from cell lysates extracted from human brain tissues samples (six each from AMC, AD 3,3, and AD 4,4 patients) were quantified using Bradford reagent (Bio-Rad; Hercules, CA, USA), and a total of 30 μg of protein aliquots from each sample were electrophoresed for 2 h at 100 V on 4–20% gradient bis-tris acrylamide gels from BioRad Life Science, Hercules, CA, USA, and transferred to a nitrocellulose membrane (Trans-Blot Turbo Transfer Pack, 0.2 μ Midi Nitrocellulose Membrane, catalog #s: 1704159, 1704159EDU) using the TRANS BLOT TURBO system. Blots were blocked with a BSA blocker for 1 h (Pierce) and incubated overnight at 4 °C, with primary antibodies listed in Table 2. After five washes of 5 min each, membranes were incubated for 1 h at RT with a 1:3000 dilution HRP-conjugated secondary antibody—goat anti-rabbit IgG (Cell Signaling Technologies, Danvers, MA, USA)—and developed using an ECL chemiluminescence detection kit (Pierce). Data were digitized and analyzed using ImageJ software version 2.0 (NIH).

The same blot was used to detect the expression levels of different proteins after stripping and reprobed with different antibodies. A stripping buffer at pH 2.2 (Glycine, Tris, and SDS with 1% Tween 20) was used. Briefly, after finishing the image acquisition, the blot was immersed with stripping buffer and incubated for 20 min with constant shaking 3 times. After stripping, the blot was washed with TBST, blocked with BSA blocker for 1 h (Pierce), and incubated overnight at 4 °C with primary antibodies.

### 2.8. Statistical Analysis

Data were analyzed using an unpaired *t*-test, and values were considered significantly different when the two-tailed *p* value was ≤0.05. Results are expressed as mean ± SEM.

## 3. Results

**Figure 1:**
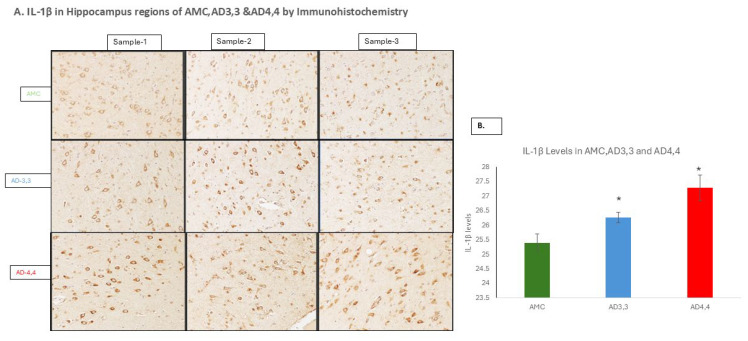

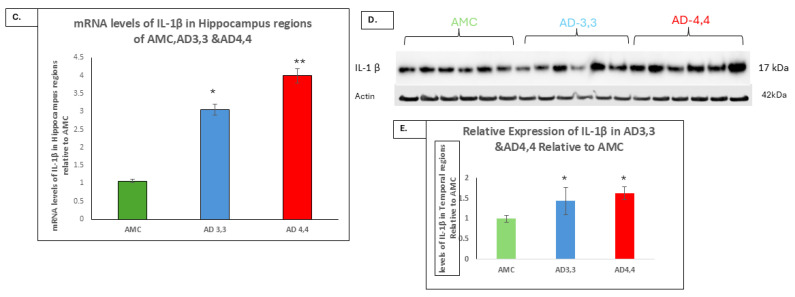
IL-1β levels are higher in hippocampus regions of Alzheimer’s brains. (**A**) Immunohistochemical detection of IL-1β in AD patients demonstrates elevation of IL-1β levels in patient carriers of both APOE *Ɛ*3/*Ɛ*3 (AD 3,3) and APOE *Ɛ*4/*Ɛ*4 (AD 4,4) compared to AMC *Ɛ*3,3. IL-1β levels are higher in hippocampus regions of Alzheimer’s brains. (**B**) Histogram showing the relative expression levels of IL-1β levels in hippocampus specimens with APOE *Ɛ*3/*Ɛ*3 (AD 3,3) and APOE *Ɛ*4/*Ɛ*4 (AD 4,4) compared to AMC *Ɛ*3,3. Histogram shows mean ± SEM. Significant differences from AMC 3,3 patients (*n* = 3) were determined by two-tailed *t*-tests within ANOVA. (**C**) The relative levels (mRNA/18S) of IL-1β transcripts determined by real-time RT-PCR. (**D**) Protein levels (IL-1β/Actin) in hippocampus specimens from AD and AMC patients, analyzed by Western blotting technique. (**E**) Histogram shows mean ± SEM. Significant differences from AMC 3,3 patients (*n* = 6) were determined by two-tailed *t*-tests within ANOVA: *p* = 0.01 for AD 3,3, and *p* = 0.01 for AD 4,4, (*n* = 6). * indicates *p* < 0.05; ** indicates *p* < 0.005, via 2-tailed *t*-test for an N of 3 biological repeats, represented as individual data points.

To more clearly define the role of neuroinflammation in Alzheimer’s neuropathogenesis, particularly as it may be influenced by the inheritance of one or both APOEε4 alleles, we assessed the roles played by IL-1β in the regulation of its downstream signaling proteins, in particular the TLR-MyD88-NFκB signaling cascade, leading to increases in Cox-1 and Cox 2 and further increases in IL-1β in a self-perpetuating cycle, more in AD 4,4 carriers than in AD 3,3 or AMC 3,3. Based on our reports showing that inheritance of the APOEε4 gene is associated with a diminution in autophagy in the brain, we hypothesized that ApoE4 influences these pathological events via elevating the production of IL-1β. Determining the expression levels of IL-1β protein in human brain tissue samples from AD 4,4 carriers for comparison to AD 3,3 and AMC 3,3, we found that the levels of IL-1β were higher in AD 4,4 brains than in either AD 3,3 or AMC brains (Figure 1A,B), as the mRNA levels of IL-1β were greater in the brains of AD 4,4 (*p*-0.0006) carriers compared to that in either AD 3,3 (*p*-0.005) or AMC carriers (Figure 1C). AD 4,4 brains had three-fold higher IL-1β expression levels (*p*-0.0006) than did AD 3,3 brains, and a 2.5-fold higher expression (*p*-0.005) compared to AMCs. Relative to AD 3,3 the levels of IL-1β protein in AD 4,4 carriers were 2.41-fold higher (*p*-0.005), which showed 1.38-fold increased expression compared to AMC homogenates (Figure 1D,E). Further, the levels of IL-1β and its signaling cascade were higher in SH-SY5Y, T98G cells expressing E4 genes than in SY5Y, T98G cells expressing E3 genes (Supplementary Figure S1A,B).

### 3.1. The Levels of an IL-1β-Driven Pathway, TLR, MyD88, and NFκB Were Higher in AD 4,4 Carriers than in Either AD 3,3 or AMC

**Figure 2:**
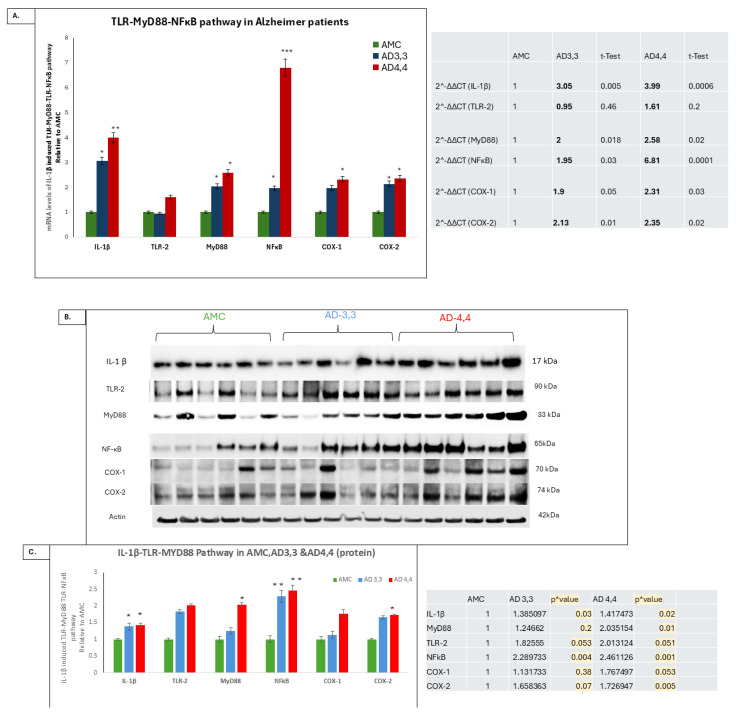
TLR-MyD88-NFκB pathway was elevated in APOE *Ɛ*4/*Ɛ*4 (AD 4,4) patients compared to APOE *Ɛ*3/*Ɛ*3 (AD 3,3) and AMC. (**A**) Relative levels (mRNA/18S) of TLR, MyD88, NFκB, COX-1, and COX2 transcripts were determined by real-time RT-PCR in hippocampus regions from AD and AMC patients, analyzed by disease state and separated by APOE genotype. (**B**) TLR-MyD88-NFκB pathway protein levels were elevated in APOE *Ɛ*4/*Ɛ*4 (AD 4,4) patients compared to APOE *Ɛ*3/*Ɛ*3 (AD 3,3) and AMC. Relative protein levels (normalized to Actin) of TLR, MyD88, NFκB, COX-1, and COX-2 were determined by Western blotting in hippocampus regions from AD and AMC patients, analyzed by disease state and separated by APOE genotype. (**C**) Histogram shows means ± SEM. Significant differences from AMC 3,3 patients (*n* = 6) were determined by two-tailed *t*-tests within ANOVA (Bonferroni-adjusted, 0.02): *p* = 0.01 for AD 3,3, (*n* = 6) and *p* = 0.01 for AD 4,4, *n* = 6. * indicates *p* < 0.05; ** indicates *p* < 0.005; and *** indicates *p* < 0.0005, via 1-tailed *t*-test for an N of 3 biological repeats, represented as individual data points.

Both mRNA (Figure 2A) and protein levels (Figure 2B,C) of TLR-2, MyD88, NFκB, COX-1, and COX-2 (Figure 2A) were significantly higher in AD 4,4 than in either AD 3,3 or AMC (Figure 2A,B). Our data showed that both AD 4,4 and AD 3,3 carriers have significantly more neuroinflammation than AMCs, with AD 4,4 having higher expression levels of neuroinflammatory markers like TLR, MyD88, and NFκB and AD 3,3 having lesser expression levels of IL-1β compared to AD 4,4, together with increased levels of IL-1β’s signaling cascade and further neuroinflammation.

### 3.2. Autophagy Was Dysregulated in APOεE4 Carriers

**Figure 3:**
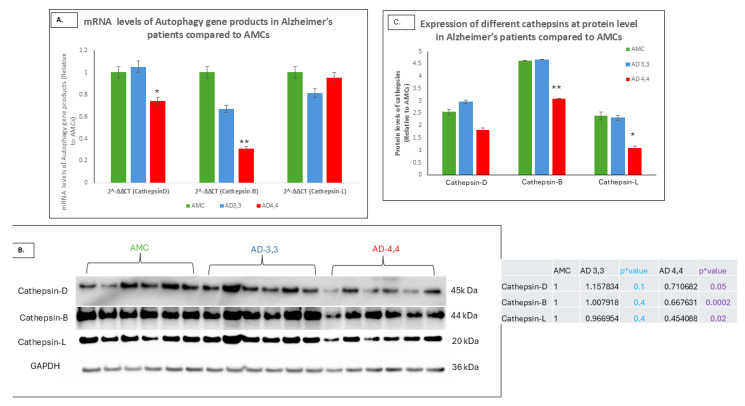
Dysregulation of autophagy by diminished cathepsins in APOE *Ɛ*4/*Ɛ*4 (AD 4,4) patients compared to APOE *Ɛ*3/*Ɛ*3 (AD 3,3) and AMC. (**A**) mRNA expression levels of cathepsin D, B, and L were determined in human hippocampus regions of APOE *Ɛ*4/*Ɛ*4 (AD 4,4) patients and compared to APOE *Ɛ*3/*Ɛ*3 (AD 3,3) and AMC patients by RT-PCR and Western blotting analysis from all the three groups (each *n* = 6). (**B**) Protein levels (cathepsin D, B, and L/GAPDH) in hippocampus specimens from APOE *Ɛ*4/*Ɛ*4 (AD 4,4) patients compared to APOE *Ɛ*3/*Ɛ*3 (AD 3,3) and AMCs, analyzed by Western blotting technique. (**C**) Histogram shows protein levels (cathepsin B, D, and L/GAPDH) mean ± SEM. Significant differences from AMC 3,3 patients (*n* = 6) were determined by two-tailed *t*-tests within ANOVA (Bonferroni-adjusted α, 0.02): *p* = 0.01 for AD 3,3, and *p* = 0.01 for AD 4,4, (*n* = 6). * Indicates *p* < 0.05; ** indicates *p* < 0.005, via 1-tailed *t*-test for an N of 3 biological repeats, represented as individual data points.

In our previous studies [7], we demonstrated that when ApoE4 binds to CLEAR DNA, it blocks the transcription of three mRNAs, SQSTM, MAP1LC3B, and LAMP2, to produce three proteins, including sequestosome p62, LC3B, and Lamp 2, each of which is necessary for lysosomal autophagy. In addition, we showed that three autophagy-related cathepsins that are also encoded on CLEAR DNA are downregulated in APOE4,4 carriers showed [20]. It can be hypothesized that this ApoE4-related diminution in the effective clearance of cellular debris in APOEε4,4 carriers contribute to an earlier onset of Alzheimer’s as well as the elevated density of Aβ plaques and neurofibrillary tangles in such carriers. Our identification and quantification of the expression levels of both mRNAs and proteins of three cathepsins, reported [21,22,23,24] to be involved in lysosomal autophagy, as measurements of the both AD 3,3 and AMC3,3 carriers had higher mRNA and protein levels than did AD 4,4 patients (Figure 3A–C), further evidence of the dramatic downregulation of lysosomal autophagy as a result of inheritance of the Alzheimer’s gene (*p* = 0.0002 for cathepsin B and *p* = 0.02 for cathepsin L, respectively). 

### 3.3. Interleukin-1β and Aβ Accumulation

**Figure 4:**
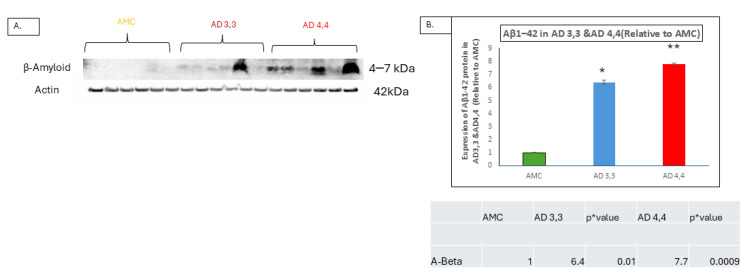
Aβ accumulation is elevated in APOE *Ɛ*4/*Ɛ*4 carriers. (**A**) Protein levels (**Aβ**/GAPDH) in hippocampus specimens from APOE *Ɛ*4/*Ɛ*4 (AD 4,4) patients compared to APOE *Ɛ*3/*Ɛ*3 (AD 3,3) and AMC patients, analyzed by Western blotting technique, compared with that of Interleukin-1β expression levels. (**B**) Histogram shows protein level (**Aβ**/GAPDH) mean ± SEM. Significant differences from AMC 3,3 patients (*n* = 6) were determined by two-tailed *t*-tests within ANOVA (Bonferroni-adjusted α, 0.02): *p* = 0.01 for AD 3,3, and *p* = 0.01 for AD 4,4, (*n* = 6). * Indicates *p* < 0.05; ** indicates *p* < 0.005, via 1-tailed *t*-test for an N of 3 biological repeats, represented as individual data points.

Aβ accumulation levels are higher in brain tissues from AD 4,4 carriers than in those from either AD 3,3 or AMC carriers (Figure 4A). AD 4,4 carriers showed an approximately eight-fold increase (*p* ≤ 0.0009) in Aβ protein over the levels in AMCs and six-fold compared to AD 3,3 carriers (*p* ≤ 0.01). This is consistent with the idea that neuroinflammation is the most significant characteristic feature of Alzheimer’s neuropathogenesis, with or without the impact of inheritance of APOE 4,4, as even AD 3,3 carriers have significant levels of Aβ accumulation, which is correlated with the expression of IL-1β, without which Aβ plaques and neurofibrillary tangles, the hallmarks of Alzheimer’s, are not produced.

### 3.4. In Vitro Treatment of NT2 Cells with ApoE4 Increased the Transcription and Production of Neurotoxic Markers, Including CD109 and PTG-(A2)

**Figure 5:**
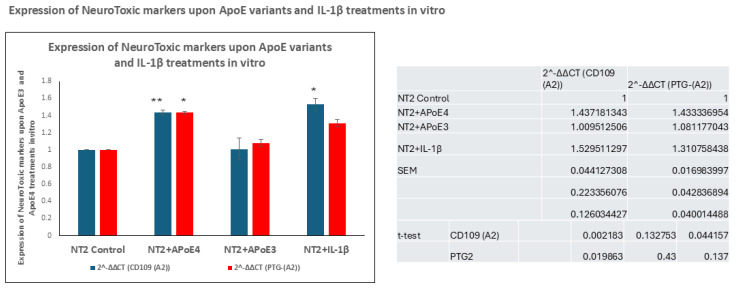
ApoE4 and IL-1β treatment elevates neurotoxic markers upon treatment in vitro. mRNA expression levels of CD109 and PTG(A)2 in NT2 cells treated with ApoE4 at 100 nM/mL and IL-1β at 100 ng/mL increased the expression of CD109 by RT-PCR. Histogram shows mRNA level (mRNA/18S)) mean ± SEM. Significant differences were determined by two-tailed *t*-tests within ANOVA (Bonferroni-adjusted α, 0.02): * indicates *p* < 0.05; ** indicates *p* < 0.005, via 1-tailed *t*-test for an N of 3 biological repeats, represented as individual data points.

LaFerla and colleagues showed that neuronal stress results in elevated levels of Aβ accumulation via overexpression of neurotoxic markers, including CD109 [25] and (PTG-(A2)), which together decrease the transforming growth factor β (TGF-β)-signaling pathway, which results in both loss of the extracellular matrix and an approximate fifty percent reduction in TGF-β expression [26]. In our experiments, ApoE4 treatment of NT2 cells at 100 nM increased the expression of CD109 by 1.4 times (*p* ≤ 0.002). Similarly, relative to untreated NT2 cells, IL-1β treatment at 30 ng/mL increased CD109 to 1.5-times (*p* ≤ 0.04) (Figure 5), whereas ApoE3 treatment did not increase CD109 compared to untreated cells. ApoE4 treatment at 100 nM increased the mRNA expression of PTG-(A2) to 1.4-X (*p* ≤ 0.01) and IL-1β at 30 ng/mL increased CD109 to 1.5 times compared to untreated NT2 cells (*p* ≤ 0.01); ApoE3 treatment did not increase PTG-(A2). This induction of elevated expression of these neurotoxic markers by ApoE4 and IL-1β identifies them as causative factors in Alzheimer’s neuropathogenesis to a significant degree due to their ability to induce expression of two toxic factors, including CD109 and PTG-(A2).

## 4. Discussion

In the face of a plethora of evidence from studies on the neuropathogenesis of AD, together with such evidence as we present here regarding the driving roles of Two Worlds in the genesis and progression of AD, it is clear that the inheritance of the Alzheimer’s gene (APOEε4, World One), which forestalls the production of three proteins that are essential for lysosomal autophagy for clearance of metabolic waste from cells and recycling of spent proteins in times of distress or deprivation, together with its prevalence, which is evident in that it is present in 50 to 65 percent of people with AD, is a large contributor to the development of Alzheimer’s [1,2,3]. However, the preeminent power of the other of the Two Worlds, the immune cytokine IL-1β, is evidenced by the fact that IL-1β drives production of the precursor protein, βAPP, which is necessary for the formation of Aβ in plaques. IL-1β is also an important factor in the development of the other cardinal feature of Alzheimer’s, viz., neurofibrillary tangles, via the driving power of IL-1β to increase the synthesis and activation state of MAPK p38 toward phosphorylation of tau [11,14], which is necessary for the formation of neurofibrillary tangles. It is curious to us that in the face of the long and well-known neuroinflammatory power of IL-1β in the genesis of both Aβ plaques and neurofibrillary tangles—especially alongside the fact that IL-1β also drives the synthesis and phosphorylation of a-synuclein, which is necessary for formation of the Lewy bodies, the bellwether of Parkinson’s disease—it remains underemphasized [19]. So far, much effort has been devoted to the clearance of Aβ plaques as a strategy to defeat or delay the onset of dementia of Alzheimer’s, explaining why the Amyloid Hypothesis, which posits that it is AB plaques, themselves, that are the cause of Alzheimer’s dementia, has persisted without an eye toward the thought of interfering in the driving power of neuroinflammation, i.e., IL-1β, the driver of the cardinal features of not only Alzheimer’s but of Parkinson’s. 

IL-1β activates the TLR2/MyD88/NF-κB pathway in Parkinson’s disease, leading to α-synuclein spreading in Parkinson’s disease (PD), multiple-system atrophy (MSA), and dementia with Lewy bodies [19]. The driving power of IL-1β in activating the neuroinflammatory pathway that we showed here is more evidence that supports the pathological role in driving Alzheimer’s pathogenesis. Targeting neuroinflammation with alogliptin, a dipeptidyl peptidase-4 inhibitor, has shown neuroprotective effects by targeting the TLR4/MYD88/NF-κB signaling cascade against lipopolysaccharide (LPS)-induced neuroinflammation and cognitive impairment in mice [27]. The long-term use of non-steroidal anti-inflammatory drugs (NSAIDs), which reduce COX-1 and COX-2, has been associated with decreased dementia risk [28]. Flurbiprofen, a well-known NSAID, exerts anti-inflammatory effects by inhibiting cyclooxygenase (COX) enzymes, particularly COX-1 and COX-2. Flurbiprofen and its R-enantiomer, R-flurbiprofen, have shown to selectively modulate the activity of gamma-secretase, an enzyme crucial for generating amyloid-beta (Aβ) peptides from the amyloid precursor protein (APP) [29]. Recently, we have shown the power of neuroinflammation, i.e., the role of IL-1β and its downstream signaling cascade, in glioblastoma pathogenesis, demonstrating targeting IL-1β and its signaling cascade using an IL-1 Receptor Antagonist as a promising therapy [30]. A recent study by Artur Shvetcov et al. 2025 published in *Nature Medicine* clearly explains the evidence of neurodegeneration because of a conserved, systemic pro-inflammatory immune proteomic signature-associated with APOE ε4 carriers [31].

Knowing all the published data regarding the driving power of IL-1β and the related inheritance of the Alzheimer gene as reported here, it is particularly curious, in the face of the ultimate failure of improvement in forestalling further cognitive decline in even one of the many human trials aimed at clearance of Aβ plaques in the human brain, that, relatively speaking, trials have neglected to aim at the power of neuroinflammation and the Alzheimer’s gene as provocateurs of the neuropathogenesis of Alzheimer’s. Recently, we reported on a study targeting ApoE4 with CBA2, a small molecule that binds exclusively to the amino acid DNA-binding sequence of ApoE4 protein that blocks ApoE4 interactions with CLEAR DNA motifs and, in this way, restores transcription of the essential genes, including SQSTM1, MAP1LC3B, and LAMP2, which are required for efficient clearance of pathognomonic aggregates via lysosomal autophagy [32]. Having in hand CBA2 for stopping downstream effects of inheritance of APOEε4, together with unpublished results regarding a newly constructed foil for IL-1β that preliminarily suggests benefit in decreasing plaque and tangle formation, we are now in position to offer these two foils against the Two World promoters of Alzheimer’s and Parkinson’s and, as we believe, other neurodegenerative diseases characterized by aggregate accumulation.

## Figures and Tables

**Table 1:** RT-PCR primers used in the study.

Gene (Direction)	Sequence	Species
MyD88	(F)5′-CCA GCA TTG AGG AGG ATT GC-3′ (R)5′-GCT CTG CTG TCC GTG GGA-3′	Human
TLR-2	(F)5′-AAG GGC AGC TCA GGA TCT TT-3′ (R)5′-AGA CTG CCC AGG GAA GAA AA-3′	Human
NF-κB p65	(F)5′-AGA TAC CAC CAA GAC CCA CC-3′ (R)5′-CTG TCC CTG GTC CTG TGT AG-3′	Human
COX-1	(F)5′-GCT GAG TGG CTA TTT CCT GC-3′ (R)5′-CTC GTA GCT GTA CTC CTG GG-3′	Human
COX-2	(F)5′-CAA TCT GGC TGA GGG AAC ACA ACA-3′ (R)5′-ATC TGC CTG CTC TGG TCA ATG GA-3′	Human
Cathepsin B	(F)5′-TCT CTG ACC GGA TCT GCA TC 3′ (R)5′-TCA CAG GGA ATG GAG TA-3′	Human
Cathepsin D	(F)5′-CAG AAG CTG GTG GAC CAG AAC-3′ (R)5′-TGC GGG TGA CAT TCA GGT AC-3′	Human
Cathepsin L	(F)5′-TAG AGG CAC AGT GGA CCA AG-3′ (R)5′-TGC GGG TGA CAT TCA GGT AG-3′	Human
18S	(F)5′-TTC GGA CGT CTG CCC TAT CAA-3′ (R)5′-ATG GTA GGC ACG GCG ACT A-3′	Human

RT-PCR conditions used in the study were 40 cycles at 95 °C for 15 s, followed by 60 °C for 60 s, and 40 °C for 60 s. All R-PCR reactions mentioned above were performed using 10 ng total RNA in 4 μL (2.5 ng/μL). mRNA levels mentioned in the manuscript are relative to the levels of 18S rRNA. Data is presented as a ΔΔCt value, which is the difference in ΔCt values between the treated sample and control sample. The expressed gene was normalized to 18 s, which is a housekeeping gene.

**Table 2:** Antibodies used in the study.

Antibody	Species	Cat#	Company
IL-1β	Mouse mAb	12242S	Cell Signaling Technology IL USA
TLR-2	Rabbit mAb	#13744	Cell Signaling Technology
MyD88	Rabbit mAb	Ab133739	Abcam
NF-κB p65	Rabbit mAb	Ab32536	Abcam
COX-1	Rabbit mAb	Ab109025	Abcam
COX-2	Rabbit mAb	Ab179800	Abcam
Cathepsin B	Rabbit mAb	3383S	Cell Signaling Technology
Cathepsin D	Rabbit mAb	740892	Cell Signaling Technology
Cathepsin L	Rabbit mAb	55914	Cell Signaling Technology
GAPDH	Mouse mAb	sc47724	Santa Cruz
β-Actin	Rabbit mAb	#4970	Cell Signaling Technology

## Data Availability

Data are contained within the article and Supplementary Materials.

## Supplementary Materials

The following supporting information can be downloaded at: https://www.mdpi.com/article/10.3390/cells14151216/s1, Figure S1. TLR-MyD88-NFκB pathway was elevated in SY5Y cells stably transformed to express APOE *Ɛ*3/*Ɛ*3 and APOE *Ɛ*4/*Ɛ4*.
