# Supplementary material for: When Two Worlds Collide: The Contribution and Association Between Genetics (APOEε4) and Neuroinflammation (IL-1β) in Alzheimer’s Neuropathogenesis
Source: Cells. 2025 Aug 7;14(15):1216. doi: 10.3390/cells14151216 (PMC12345965; doi:10.3390/cells14151216)
Supplement: Supplementary file 1 [file cells-14-01216-s001.zip › cells-3801986-supplementary.pdf]

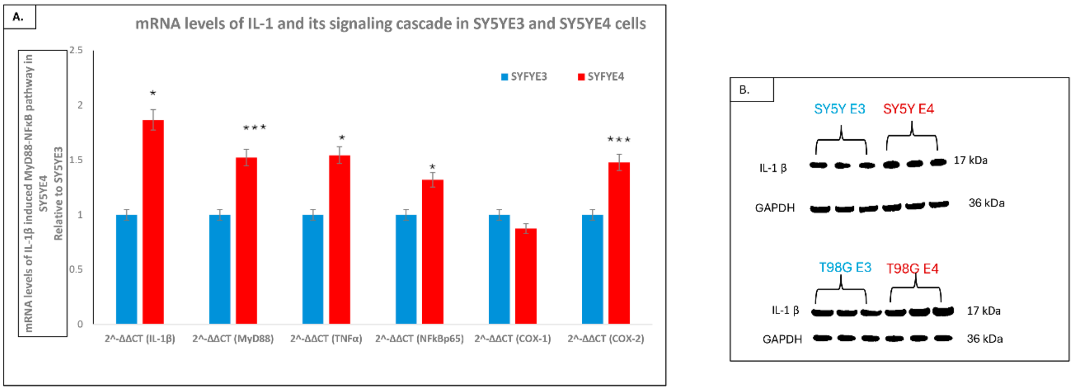

Figure S1. **TLR-MyD88-NFκB pathway was elevated in SY5Y cells stably transformed to express APOE Ɛ3/ Ɛ3 and APOE Ɛ4/ Ɛ4.** (A)Relative levels (mRNA/18S) of TLR, MyD88, NFκB, COX-1 and COX2 transcripts were determined by real-time RT-PCR in SY5Y cells stably transformed to express APOE Ɛ3/ Ɛ3 and APOE Ɛ4/ Ɛ4 APOE genotype. Histogram shows means ± SEM. Significance of differences was determined by two-tailed t-tests within ANOVA (Bonferroni-adjusted, 0.02): P= .01 for AD 3,3, (n = 5) and P=.01 for AD 4,4, n= 6. * Indicates $p < 0.05$, ** indicates $p < 0.005$ *** indicates $p < 0.0005$, via 1-tailed t test for an N of 3 biological repeats, represented as individual data points.

(B) Protein levels of IL-1β were higher in SY5Y and T98G cells stably transformed with APOE Ɛ4/ Ɛ4 than APOE Ɛ3/ Ɛ3 (IL-1β/GAPDH).
